# The number of examinations required for the accurate prediction of the progression of the central 10-degree visual field test in glaucoma

**DOI:** 10.1038/s41598-022-23604-z

**Published:** 2022-11-07

**Authors:** Takashi Omoto, Ryo Asaoka, Tadamichi Akagi, Akio Oishi, Manabu Miyata, Hiroshi Murata, Yuri Fujino, Kazunori Hirasawa, Tatsuya Inoue, Masaki Tanito, Nobuyuki Shoji

**Affiliations:** 1 Department of Ophthalmology, University of Tokyo Graduate School of Medicine, 7-3-1 Hongo, Bunkyo-Ku, Tokyo, 113-8655 Japan; 2 Department of Ophthalmology, Seirei Hamamatsu General Hospital, Shizuoka, Japan; 3 Seirei Christopher University, Shizuoka, Japan; 4 Nanovision Research Division, Research Institute of Electronics, Shizuoka University, Shizuoka, Japan; 5 The Graduate School for the Creation of New Photonics Industries, Shizuoka, Japan; 6 Department of Ophthalmology and Visual Sciences, Kyoto University Graduate School of Medicine, Kyoto, Japan; 7 Division of Ophthalmology and Visual Science, Niigata University Graduate School of Medical and Dental Sciences, Niigata, Japan; 8 Department of Ophthalmology and Visual Sciences, Nagasaki University, Nagasaki, Japan; 9 Center Hospital of the National Center for Global Health and Medicine, Tokyo, Japan; 10 Department of Ophthalmology, Shimane University Faculty of Medicine, Shimane, Japan; 11 Department of Ophthalmology, School of Medicine, Kitasato University, Kanagawa, Japan; 12 Department of Ophthalmology and Micro-Technology, Yokohama City University School of Medicine, Kanagawa, Japan

**Keywords:** Eye diseases, Glaucoma

## Abstract

The purpose of the study was to investigate the number of examinations required to precisely predict the future central 10-degree visual field (VF) test and to evaluate the effect of fitting non-linear models, including quadratic regression, exponential regression, logistic regression, and M-estimator robust regression model, for eyes with glaucoma. 180 eyes from 133 open angle glaucoma patients with a minimum of 13 Humphrey Field Analyzer 10-2 SITA standard VF tests were analyzed in this study. Using trend analysis with ordinary least squares linear regression (OLSLR), the first, second, and third future VFs were predicted in a point-wise (PW) manner using a varied number of prior VF sequences, and mean absolute errors (MAE) were calculated. The number of VFs needed to reach the minimum 95% confidence interval (CI) of the MAE of the OLSLR was investigated. We also examined the effect of applying other non-linear models. When predicting the first, second, and third future VFs using OLSLR, the minimum MAE was obtained using VF1–12 (2.15 ± 0.98 dB), VF1–11 (2.33 ± 1.10 dB), and VF1–10 (2.63 ± 1.36 dB), respectively. To reach the 95% CI of these MAEs, 10, 10, and 8 VFs were needed for the first, second and third future VF predictions, respectively. No improvement was observed by applying non-linear regression models. As a conclusion, approximately 8–10 VFs were needed to achieve an accurate prediction of PW VF sensitivity of the 10-degree central VF.

## Introduction

Glaucoma is a major cause of blindness and vision impairment worldwide^1–3^, and visual field (VF) tests are essential to monitor the progression of the disease^4,5^. Accurate assessment of VF progression is important in glaucoma, because inaccurate assessment can lead to overtreatment and undertreatment. The overtreatment can lead to unnecessary complications treatment because the treatment involves a reduction in the intraocular pressure through medical and/or surgical interventions^6–10^ and these are associated with various ocular and general complications^11–15^. The undertreatment may not stop the progression of the disease. VF sensitivity fluctuates in the short^16^ and long terms^17^, measurement noise is considerable even with good reliability indices^18,19^, and the reliability of measured VF is inherently affected by the patient’s concentration. The ability of VF trend analyses to accurately evaluate and predict the progression of VF is significantly affected by VF variability and the number of VFs, in particular point-wise (PW) linear regression (PLR)^20^. Therefore, the number of VFs and the reliability of PLR results have been widely discussed^21,22^. There are a number of procedures that have been used to evaluate visual field progression and predictive ability^23^. Chauhan et al.^24^ reported the number of VF tests required to detect significant MD progression, titrated by number of tests performed per time period and variability. We previously investigated this issue using Humphrey Field Analyzer (HFA; Carl Zeiss Meditec AG, Dublin, CA, USA) 24-2 tests; as a result, approximately 10 VFs were needed to achieve an accurate prediction of PW VF sensitivity, where the reliability of the trend analysis was estimated by the prediction accuracy^25^. Because the variance in VF sensitivity in the central area is considerably different from (i.e., much smaller than) that in the peripheral area^26^, different results could be obtained between the HFA 24-2 and 10-2 tests.

More than 30% of retinal ganglion cells residing in the central VF region correspond to the HFA 10-2 test^27^, whereas only 4 points are allocated in the HFA 24-2 test. Although 10-2 test cannot detect early glaucomatous visual field defects such as nasal step, several studies have suggested the importance of HFA 10-2 tests, in which 68 points are placed 2 degrees apart in the same region^28–33^. In addition, recent studies have suggested that the sensitivity in the central area such as that of the HFA 10-2 test is no less important than that of the HFA 24-2 test, in particular when assessing the vision-related quality of life in patients with glaucoma^34,35^, although it has still remained controversial^33^. Therefore, the aim of this study was to investigate the number of examinations required for the accurate prediction of the central 10-degree VF test.

Furthermore, many studies have investigated the prediction performance of various linear and non-linear regression models. We previously reported that there was no significant merit to using these models over OLSLR to predict the HFA 24-2 test^25^. The second purpose of the current study was to evaluate the effect of these models using the HFA 10-2 test in the present study.

## Results

The demographic details of the 180 included eyes are summarized in Table 1. 85 eyes were right eyes, and the remaining 95 eyes were left eyes. The mean ± standard deviation (SD) of age and the MD at the initial examination were 56.1 ± 11.4 years and − 19.8 ± 8.1 dB, respectively. The total duration of follow-up and the MD slope during the study period were 7.5 ± 2.0 years and − 0.33 ± 0.40 dB/year, respectively. When predicting the first, second, and third future VFs using OLSLR, the minimum absolute prediction error was obtained using VF1–12 (2.15 ± 0.98 dB), VF1–11 (2.33 ± 1.10 dB), and VF1–10 (2.63 ± 1.36 dB), respectively.

**Table 1:** Demographics of the study subjects.

Variables	Values	Median (IQR)
Eyes, R: L	85/95	
Age at the initial VF, mean ± SD, y	56.1 ± 11.4	57 (49, 65)
MD at the initial VF, mean ± SD, dB	− 19.8 ± 8.1	− 21.6 (− 25.7, − 14.1)
MD at the 5th VF, mean ± SD, dB	− 20.5 ± 8.0	− 22.2 (− 26.5, − 14.4)
MD at the 10th VF, mean ± SD, dB	− 21.4 ± 7.9	− 23.2 (− 27.3, − 15.2)
MD at the 13th VF, mean ± SD, dB	− 22.2 ± 7.8	− 23.5 (− 27.9, − 16.4)
Time from 1st to 5th examination, mean ± SD, years	2.6 ± 1.4	2.1 (1.8, 2.8)
Time from 1st to 10th examination, mean ± SD, years	5.6 ± 1.8	5.2 (4.5, 6.5)
Time from 1st to 13th examination, mean ± SD, years	7.5 ± 2.0	7.1 (6.2, 8.4)

*VF* visual field, *MD* mean deviation, *SD* standard deviation, *IQR* interquartile range.

**Figure 1:**
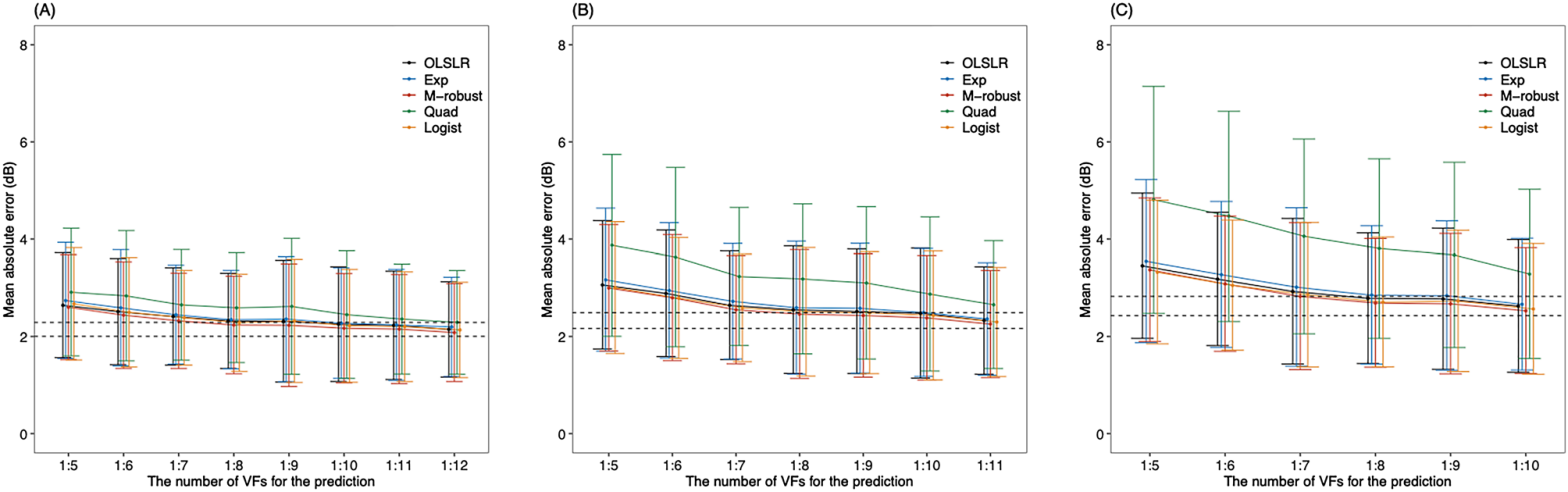
MAE values in the first (**A**), second (**B**) and third (**C**) future PW VF prediction with each formula. Dashed line shows the minimum 95% confidence interval of the MAE of the OLSLR. MAE: mean absolute error, PW: point-wise, VF: visual field, OLSLR: ordinary least squares linear regression, Exp: exponential regression, M-robust: M-estimator robust linear regression, Quad: quadratic regression, Logist: logistic regression.

Figure 1A shows the mean absolute error (MAE) for the first future VF prediction with each model. With OLSLR, ten VFs were needed for the MAE value to reach the minimum 95% confidence interval (CI) when predicting thirteenth VF. The MAEs associated with OLSLR decreased with an increase in the number of VFs used in the prediction. The MAE values in the exponential, M-robust, and logistic models were not significantly different from those with OLSLR (from the sixth to thirteenth VF predictions, Supplemental Table 1). The MAEs of the quadratic model were not significantly different from those with OLSLR (from the eleventh to the thirteenth VF predictions), but were otherwise significantly larger than those with OLSLR (from the sixth to the tenth VF predictions).

Figure 1B shows the MAEs of the second future VF prediction using each model. Similar to the first VF prediction, the MAEs associated with OLSLR decreased with an increase in the number of VFs used in the prediction, and 10 VFs were needed for the MAE value to reach the 95% CI when predicting the twelfth VF using the OLSLR. The MAE values in the exponential, M-robust, and logistic models were not significantly different from those with OLSLR (from the seventh to the thirteenth VF predictions, Supplemental Table 2). The MAEs of the quadratic model were significantly larger than those with OLSLR (from the seventh to the thirteenth VF predictions).

Figure 1C shows the MAEs of the third future VF prediction using each model. Eight VFs were needed for the MAE value to reach the 95% CI when predicting the eleventh VF using the OLSLR. The MAE values in the exponential, M-robust, and logistic models were not significantly different from those in the OLSLR (from the eighth to the thirteenth VF predictions, Supplemental Table 3). The MAEs of the quadratic model were significantly larger than those in the OLSLR (from the eighth to thirteenth VF predictions).

**Figure 2:**
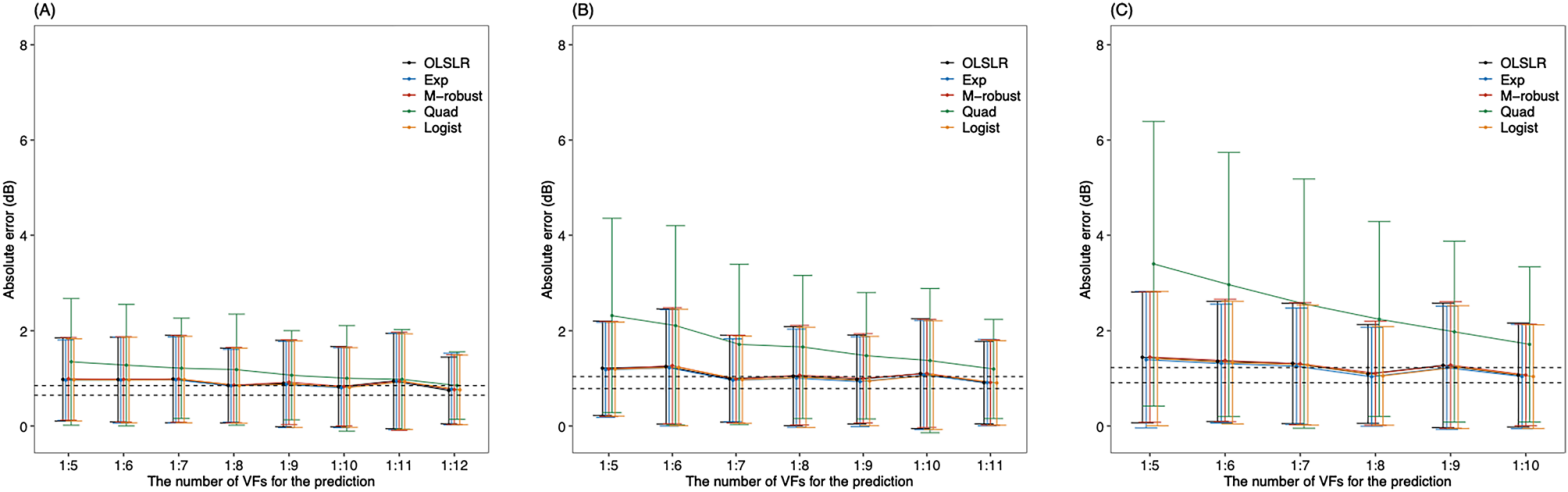
AE values in the first (**A**), second (**B**) and third (**C**) future MS prediction with each formula. Dashed line shows the minimum 95% confidence interval of the AE of the OLSLR. AE: absolute error, MS: mean sensitivities, OLSLR: ordinary least squares linear regression, Exp: exponential regression, M-robust: M-estimator robust linear regression, Quad: quadratic regression, Logist: logistic regression.

As shown in Fig. 2A, B and C, the minimum absolute error (AE) associated with OLSLR of mean sensitivity (MS) was obtained using 1) VF1–12 (0.75 ± 0.70 dB) when predicting the first future VF; 2) VF1–11 (0.91 ± 0.87 dB) when predicting the second future VF; and 3) VF1–10 (1.07 ± 1.09 dB) when predicting the third future VF. There were no significant differences in the AE associated with OLSLR, exponential regression, and M-robust models at any time points. The MAEs in the quadratic regression model were significantly worse than those of the OLSLR method at the sixth to ninth and eleventh VF prediction in the first future prediction, (Supplemental Table 4) the seventh to eleventh and thirteenth VF prediction in the second future prediction, (Supplemental Table 5) and the eighth to thirteenth prediction in the third future prediction (Supplemental Table 6).

**Figure 3:**
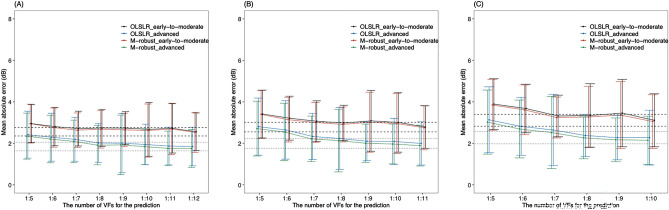
MAE values in the first (**A**), second (**B**) and third (**C**) future PW VF prediction with each formula in the early-to-moderate and advanced glaucoma groups. Dashed and dotted line shows the minimum 95% confidence interval of the MAE of the OLSLR in early-to-moderate and advanced group, respectively. MAE: mean absolute error, PW: point-wise, VF: visual field, OLSLR: ordinary least squares linear regression, Exp: exponential regression, M-robust: M-estimator robust linear regression, Quad: quadratic regression, Logist: logistic regression.

**Figure 4:**
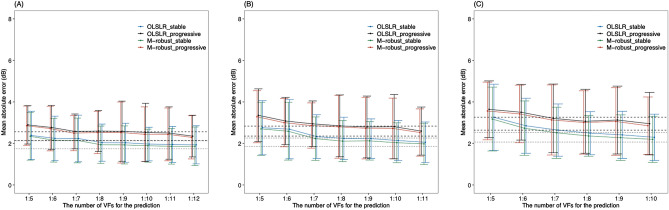
MAE values in the first (**A**), second (**B**) and third (**C**) future PW VF prediction with each formula in the stable and progressive groups. Dashed and dotted line shows the minimum 95% confidence interval of the MAE of the OLSLR in progressive and stable group, respectively. MAE: mean absolute error, PW: point-wise, VF: visual field, OLSLR: ordinary least squares linear regression, Exp: exponential regression, M-robust: M-estimator robust linear regression, Quad: quadratic regression, Logist: logistic regression.

Furthermore, same PW predictions were performed dividing by the sub-groups. Figure 3 (early-to-moderate group: 76 eyes, advanced group: 104 eyes), Fig. 4 (stable group: 89 eyes, progressive group: 91 eyes) show the changes in the MAE. There were no significant differences between the MAE using the OLSLR and M-robust methods in the first (Supplemental Table 7), second (Supplemental Table 8), and third (Supplemental Table 9) future prediction at any time points.

## Discussion

In the current study, the number of examinations required for the precise prediction of central 10-degree VF tests was investigated in 180 eyes from 133 patients with open angle glaucoma. As a result, eight (third future VF prediction) or ten (first and second future VF predictions) VFs were needed to saturate the prediction accuracy. As expected from the tendency toward smaller variance of VF sensitivity in the central area compared to the peripheral area these numbers were smaller than those in our previous study, in which the HFA 24-2 test was used (11, 10, and 9 VFs were needed for the first, second, and third future VF predictions, respectively^25^). Nonetheless, the difference was only a single VF, which suggests that both the HFA 10-2 test along with the HFA 24-2 test should be measured, at a similar frequency. This aligns with our previous studies^40,41^; the prediction accuracy of trend analysis is dramatically improved by applying LASSO regression, in which the sum of the absolute values of the regression coefficients is penalized. The magnitude of the penalty should be large when the number of VFs used in the prediction is small in both the HFA 24-2 and 10-2 tests; otherwise, the prediction accuracies are poor. Recent studies reporting the clinical importance of the HFA 10-2 test^34,35^ would further postulate this recommendation. This implies that clinicians should be careful when interpreting VF trend analysis results that use only a small number of HFA 10-2 tests.

The progression rate of MD in the HFA 10-2 should vary depending on the studied population and the disease conditions. David et al. reported that the MD slope differed with or without disc hemorrhage (− 0.50 and − 0.15 dB/year, respectively)^42^, while Kim et al. reported that the MD slope of eyes with primary open angle glaucoma (POAG) was − 0.11 dB/year, while that with normal tension glaucoma was − 0.33 dB/year^43^ in their prospective studies. On the other hand, Wang et al. reported that the average MD slope was − 0.37 dB/year in a recent retrospective study^44^. De Moraes et al.^45^ reported the median rate of the 10–2 MD change was − 0.38 dB/year in a retrospective study. The mean MD slope in our study (− 0.33 ± 0.40 dB/year) is comparable to these past studies, despite the relatively worse initial MD value (− 19.8 ± 8.1 dB).

Similar to our previous work with the HFA 24-2 test^25^, in the current study there was no benefit observed by using the exponential, quadratic, or logistic models over OLSLR both in the PW and MS analyses. One of the differences between these studies was that, in the previous study, the prediction accuracy tended to be smaller, with the M-estimator robust linear regression model compared to OLSLR, although they were not significantly different. A similar tendency was observed in the current study, including sub-analysis divided by sub-groups. In the M-estimation, the weights of those with large residuals are reduced using a specific function, and the progression slope is estimated using the weighted least squares method. The different tendencies between the previous and current studies may be attributed to the smaller variance in VF sensitivity in the central area than that in the peripheral area, which masked the merit of the mechanism of the noise reduction in the M-estimation. Chen et al. reported that exponential and logistic models enabled more accurate prediction^46^; however, this finding was not observed in the current study. Similar results were also observed in our previous study using the HFA 24-2 test. The reason for these contradicting results may be attributed to differences in the analysis method; in the study by Chen et al. models fitted to the first 5 years of VF data were used to predict the VF measurements at 1, 2, 3, and 5 years after the last VF was used to estimate the model parameters.

Despite our results, in the clinic, it is time-consuming and costly to carry out a 10-2 VF test in addition to a central 24-degree VF test. Indeed, measuring the central 24-degree VF testing with sufficient frequency may be beyond the reality of busy clinics^22,47,48^. This implies that complementing the HFA 10-2 trend analysis using other measurements would be clinically useful. For instance, we previously reported a method to estimate the MD of the HFA 10-2 test from the HFA 24-2 test, which resulted in improved accuracy of the MD trend analysis of HFA 10-2, in particular when the number of HFA 10-2 tests is small^49^. Furthermore, we have suggested the possibility of estimating the HFA 10-2 test using the results of optical coherence tomography^50–52^. Nonetheless, the prediction accuracies of these models are not at the clinical level (5.5 dB at the best)^52^. Another possible approach to overcome this problem is to cluster the VF into small sectors; a compromise method between trend analysis could use a value reflecting total area sensitivities, such as MD and PW linear regression^45,53,54^. We also previously proposed a new clustering map using an unsupervised machine leaning method^53^ and reported a favorable prediction accuracy of this method in the HFA 24–2 SITA standard^55,56^ and the 10-2^57^. However, even with this method, the prediction accuracy was relatively low when small numbers of VFs were examined^56,57^. Further investigation is needed to find ways to avoid the frequent measurements needed for the HFA 10-2 test.

There are several limitations in this study. The study population mainly consisted of severe cases; the mean MD was − 19.8 dB despite the relatively young age (56.1 years on average at the initial examination). Further investigation should be carried out using VFs at earlier stages. When analyzing progression by linear regression, it is necessary to consider not only the slope value but also the combination of the slope and p-value, and this point was not taken into account in this study. The present study was to analyze the relationship between the number of visual fields and prediction accuracy for a fixed baseline, and not for the case of changing the baseline. In many countries, using both the 24-2 and 10-2 test procedures during the same visit is tedious. Recently, a new procedure (24-2C) has been introduced, which adds 10 test locations to the 24-2 test procedure. It should be investigated in the future study whether similar results can be obtained with 24-2C. Similarly, a further study should be conducted using other algorithms of SITA FAST/FASTER. These ones have been known to be faster than SITA standard with comparable accuracy^58–60^, although this was beyond the scope of the current study. The limited usefulness of the reliability indices and possible delay of the detection time due to the information loss are recognized. This may have some influence on the current result. The more variance of VF tests can lead to the worse prediction accuracy^20^. So it is important to look at the variance of the VF, but since the usefulness of the reliability indices is limited^18,19,61–65^, it is not possible to estimate all of the impact of the variation of the VF on the prediction accuracy in each individual. VFs were measured every 6 months in average, which is the standard clinical practice in Japan. VFs were measured every 6 months in average, which is the standard clinical practice in Japan. Early detection of the VF progression can be achieved by clustering VF measurement at the beginning and end of the monitoring period^66^, however this approach is not applicable to the current study, because the current data are derived from real world clinic which never ends, unlike randomized clinical trials.

In conclusion, approximately 10 VFs were needed to achieve an accurate prediction of PW VF sensitivity of the 10-degree central VF. The application of non-linear regression models did not improve the prediction accuracy. These results suggest that it is ideal to perform the HFA 10-2 test along with HFA 24-2 test at a similar frequency.

## Methods

This study was approved by the research ethics committee of the Graduate School of Medicine and the Faculty of Medicine at the University of Tokyo, Shimane University, Kitasato University and Kyoto University. All patients provided written consent for their information to be stored in the hospital database and to be used for research. Patient consent to participate in this study was waived, and an opt-out approach was used according to the Ethical Guidelines for Medical and Health Research Involving Human Subjects presented by the Ministry of Education, Culture, Sports, Science, and Technology in Japan. Patients and the public were not involved in the design, conduct, reporting, or dissemination plans of our research. This study was performed according to the tenets of the Declaration of Helsinki.

### Participants

Participants were retrospectively recruited at the glaucoma clinics of the above-mentioned institutions. POAG patients with at least 13 reliable HFA 10-2 examinations were included in the study. An unreliable VF was defined as more than 20% fixation losses or more than 15% false-positive errors, following the manufacturer’s recommendation. Only the patient’s initial 13 VFs were analyzed when a patient had more than 13 VF test results. Cases with any ophthalmological surgical intervention during the follow-up period such as cataract and/or glaucoma surgeries were excluded from the study. Patients with other ocular diseases that could affect VF sensitivity, such as diabetes mellitus retinopathy, corneal opacity, and macular degeneration, were excluded. Patients with cataracts other than clinically insignificant senile cataracts were excluded. 180 eyes from 133 open angle glaucoma patients were included in the final analysis.

### Statistical analysis

As the first future VF prediction, using the first 5 VFs, the PW VF sensitivities of the first future (sixth) VF was predicted, and the MAE between the predicted and actual PW sensitivities was calculated; this was iterated to predict up to the thirteenth VF using the first 12 VFs. Since the prediction accuracy of visual field by 2-4 tests was significantly worse than 5 tests in our previous study, we started the prediction with 5-test series. Similar analyses were performed to predict the second future VFs (starting from the prediction of the seventh VF using the first 5 VFs, up to the prediction of the thirteenth VF prediction using the first 11 VFs), and the third future VF (starting from the prediction of the eighth VF using the first 5 VFs, up to the prediction of the thirteenth VF using the first 10 VFs). In addition to the PW prediction, the MS of the total area were also predicted in the same way, and the AE was calculated. When predicting the future VF sensitivities, the following five models were adopted in accordance with our previous report^25^:

1. OLSLR: $$y = ax + b$$
2. Exponential regression: $$y = e^{ax + b}$$
3. Quadratic regression: $$y = ax^{2} + bx + c$$
4. M-estimator robust linear regression^68^:
  $$y_{i} = \beta_{0} + \beta_{1} x_{i1} + \beta_{2} x_{i2} + \cdots \beta_{k} x_{ik} + \varepsilon_{i} = \beta x_{i} + \varepsilon_{i}$$

for the *i*th of n observations, the general M-estimator minimizes the objective function:

$\mathop \sum \limits_{i = 1}^{n} \rho \left( {\varepsilon_{i} } \right) = \mathop \sum \limits_{i = 1}^{n} \rho \left( {y_{i} - \beta x_{i} } \right)$, where the function ρ gives the contribution of each residual to the objective function.

5. Logistic regression: $y = \frac{1}{{1 + e^{ax + b} }}$, where y is the sensitivities divided by 40 to convert the values to between 0 and 1.

In all formulas, y represents the PW VF sensitivity, x represents the time from the initial VF, and a, b, and c are the model parameters to be estimated.

Following this, the minimum numbers of VFs required to reach the minimum 95% CI of OLSLR with the longest VF series (smallest MAEs) were identified for all of the first, second, and third future VF predictions. The MAEs of each model were compared using a linear mixed model approach whereby the random effect was subject. The linear mixed model adjusts for the hierarchical structure of the data, modeling how the measurements are grouped within each subject to reduce the possible bias of including both eyes from one patient^70,71^. Benjamini and Hochberg’s^72^ method was used to adjust for multiple comparisons. Statistical significance was set at 0.05. All analyses were performed using R software v.4.0.4 (The R Foundation for Statistical Computing, Vienna, Austria).

As sub-analyses, same analyses were performed in each of the following sub-groups: early-to-moderate and advanced glaucoma (initial mean deviation [MD] > − 20 dB and < − 20 dB, respectively) and stable and progressive glaucoma (MD slope > − 0.25 dB/year and < − 0.25 dB/year, respectively).

## Supplementary Information

Supplementary Information.

## Data Availability

The datasets used and analyzed during the current study available from the corresponding author on reasonable request.
