# Supplementary material for: The number of examinations required for the accurate prediction of the progression of the central 10-degree visual field test in glaucoma
Source: Sci Rep. 2022 Nov 7;12:18843. doi: 10.1038/s41598-022-23604-z (PMC9640563; doi:10.1038/s41598-022-23604-z)
Supplement: Supplementary file 1 — Supplementary Information. [file 41598_2022_23604_MOESM1_ESM.pdf]

Supplemental Table 1. The MAEs of first future VF prediction with each model.

Predicted VF	6	7	8	9	10	11	12	13
The number of VFs for the prediction	1-5	1-6	1-7	1-8	1-9	1-10	1-11	1-12
OLSLR	2.64 ± 1.08	2.51 ± 1.09	2.41 ± 1.00	2.32 ± 0.98	2.31 ± 1.25	2.25 ± 1.18	2.23 ± 1.12	2.15 ± 0.98
Exp	2.74 ± 1.19	2.59 ± 1.19	2.45 ± 1.02	2.35 ± 1.01	2.36 ± 1.29	2.27 ± 1.14	2.24 ± 1.14	2.20 ± 1.02
M-robust	2.60 ± 1.08	2.44 ± 1.10	2.32 ± 0.98	2.24 ± 1.01	2.23 ± 1.26	2.17 ± 1.13	2.15 ± 1.12	2.08 ± 1.01
Quad	2.91 ± 1.31	2.84 ± 1.34	2.65 ± 1.14	2.59 ± 1.13	2.62 ± 1.40	2.45 ± 1.31	2.36 ± 1.13	2.29 ± 1.07
Logist	2.67 ± 1.15	2.50 ± 1.13	2.39 ± 0.98	2.28 ± 1.00	2.32 ± 1.27	2.22 ± 1.16	2.20 ± 1.13	2.13 ± 0.98
OLSLR vs Exp	0.27	0.26	0.65	0.73	0.59	0.79	0.91	0.40
OLSLR vs M-robust	0.59	0.26	0.29	0.37	0.25	0.48	0.60	0.36
OLSLR vs Quad	0.0014*	<0.001*	0.0029*	0.0027*	<0.001*	0.060	0.36	0.070
OLSLR vs Logist	0.68	0.82	0.70	0.65	0.92	0.74	0.82	0.83
M-robust vs Exp	0.10	0.033*	0.12	0.27	0.10	0.37	0.60	0.12
M-robust vs Quad	<0.001*	<0.001*	<0.001*	<0.001*	<0.001*	0.012*	0.18	0.018*
M-robust vs Logist	0.41	0.38	0.42	0.65	0.25	0.66	0.80	0.40
Exp vs Quad	0.065	0.0015*	0.013*	0.0091*	0.0018*	0.10	0.42	0.33
Exp vs Logist	0.41	0.23	0.42	0.54	0.59	0.66	0.82	0.36
Quad vs Logist	0.0064*	<0.001*	<0.001*	0.0010*	<0.001*	0.025*	0.29	0.07

MAE: mean absolute error, VF: visual field, OLSLR: ordinary least squares linear regression, Exp: exponential regression, M-robust: M-estimator robust linear regression, Quad: quadratic regression, Logist: logistic regression, * represents $p < 0.05$

Supplemental Table 2. The MAEs of second future VF prediction with each model.

Predicted VF	7	8	9	10	11	12	13
The number of VFs for the prediction	1-5	1-6	1-7	1-8	1-9	1-10	1-11
OLSLR	3.06 ± 1.32	2.89 ± 1.30	2.64 ± 1.12	2.55 ± 1.31	2.52 ± 1.28	2.48 ± 1.34	2.33 ± 1.10
Exp	3.17 ± 1.47	2.95 ± 1.39	2.72 ± 1.19	2.59 ± 1.37	2.58 ± 1.34	2.49 ± 1.32	2.36 ± 1.16
M-robust	3.00 ± 1.30	2.80 ± 1.30	2.55 ± 1.11	2.46 ± 1.32	2.43 ± 1.27	2.38 ± 1.28	2.26 ± 1.10
Quad	3.87 ± 1.87	3.63 ± 1.84	3.23 ± 1.42	3.18 ± 1.54	3.10 ± 1.57	2.87 ± 1.58	2.65 ± 1.31
Logist	3.00 ± 1.35	2.79 ± 1.24	2.59 ± 1.11	2.51 ± 1.32	2.49 ± 1.25	2.43 ± 1.33	2.30 ± 1.12
OLSLR vs Exp	0.28	0.50	0.40	0.56	0.61	0.89	0.63
OLSLR vs M-robust	0.48	0.29	0.34	0.33	0.51	0.58	0.41
OLSLR vs Quad	<0.001*	<0.001*	<0.001*	<0.001*	<0.001*	0.0011*	<0.001*
OLSLR vs Logist	0.51	0.29	0.56	0.56	0.75	0.71	0.63
M-robust vs Exp	0.067	0.10	0.074	0.14	0.24	0.57	0.24
M-robust vs Quad	<0.001*	<0.001*	<0.001*	<0.001*	<0.001*	<0.001*	<0.001*
M-robust vs Logist	0.94	0.93	0.63	0.56	0.61	0.71	0.63
Exp vs Quad	<0.001*	<0.001*	<0.001*	<0.001*	<0.001*	0.0026	0.0014*
Exp vs Logist	0.067	0.073	0.16	0.34	0.51	0.71	0.50
Quad vs Logist	<0.001*	<0.001*	<0.001*	<0.001*	<0.001*	<0.001*	<0.001*

MAE: mean absolute error, VF: visual field, OLSLR: ordinary least squares linear regression, Exp: exponential regression, M-robust: M-estimator robust linear regression, Quad: quadratic regression, Logist: logistic regression, * represents $p < 0.05$

Supplemental Table 3. The MAEs of third future VF prediction with each model.

Predicted VF	8	9	10	11	12	13
The number of VFs for the prediction	1-5	1-6	1-7	1-8	1-9	1-10
OLSLR	3.45 ± 1.49	3.18 ± 1.37	2.93 ± 1.50	2.79 ± 1.34	2.77 ± 1.45	2.63 ± 1.36
Exp	3.55 ± 1.68	3.27 ± 1.50	3.02 ± 1.63	2.85 ± 1.42	2.84 ± 1.53	2.66 ± 1.36
M-robust	3.37 ± 1.47	3.08 ± 1.39	2.83 ± 1.51	2.69 ± 1.32	2.67 ± 1.44	2.53 ± 1.29
Quad	4.81 ± 2.33	4.47 ± 2.16	4.06 ± 2.00	3.81 ± 1.85	3.68 ± 1.90	3.29 ± 1.74
Logist	3.32 ± 1.48	3.05 ± 1.34	2.86 ± 1.49	2.71 ± 1.33	2.73 ± 1.45	2.57 ± 1.34
OLSLR vs Exp	0.37	0.39	0.38	0.58	0.69	0.65
OLSLR vs M-robust	0.37	0.36	0.26	0.47	0.52	0.29
OLSLR vs Quad	<0.001*	<0.001*	<0.001*	<0.001*	<0.001*	<0.001*
OLSLR vs Logist	0.19	0.25	0.39	0.54	0.69	0.49
M-robust vs Exp	0.092	0.093	0.049*	0.24	0.31	0.17
M-robust vs Quad	<0.001*	<0.001*	<0.001*	<0.001*	<0.001*	<0.001*
M-robust vs Logist	0.59	0.76	0.68	0.85	0.69	0.65
Exp vs Quad	<0.001*	<0.001*	<0.001*	<0.001*	<0.001*	<0.001*
Exp vs Logist	0.020	0.046	0.081	0.27	0.52	0.29
Quad vs Logist	<0.001*	<0.001*	<0.001*	<0.001*	<0.001*	<0.001*

MAE: mean absolute error, VF: visual field, OLSLR: ordinary least squares linear regression, Exp: exponential regression, M-robust: M-estimator robust linear regression, Quad: quadratic regression, Logist: logistic regression, * represents $p < 0.05$

Supplemental Table 4. The AEs of first future MS prediction with each model.

Predicted VF	6	7	8	9	10	11	12	13
The number of VFs for the prediction	1-5	1-6	1-7	1-8	1-9	1-10	1-11	1-12
OLSLR	0.98 ± 0.87	0.98 ± 0.89	0.99 ± 0.92	0.85 ± 0.79	0.89 ± 0.91	0.82 ± 0.84	0.94 ± 1.00	0.75 ± 0.70
Exp	0.96 ± 0.84	0.96 ± 0.91	0.97 ± 0.9	0.84 ± 0.76	0.87 ± 0.91	0.8 ± 0.84	0.94 ± 1.02	0.77 ± 0.75
M-robust	0.99 ± 0.87	0.98 ± 0.9	0.99 ± 0.91	0.86 ± 0.79	0.92 ± 0.89	0.83 ± 0.83	0.94 ± 1.03	0.76 ± 0.73
Quad	1.35 ± 1.33	1.28 ± 1.28	1.21 ± 1.06	1.18 ± 1.17	1.07 ± 0.94	1.00 ± 1.11	0.98 ± 1.05	0.85 ± 0.71
Logist	0.97 ± 0.86	0.97 ± 0.9	0.97 ± 0.91	0.85 ± 0.79	0.88 ± 0.91	0.81 ± 0.84	0.93 ± 1.00	0.76 ± 0.73
OLSLR vs Exp	0.88	0.97	0.96	0.97	0.83	0.90	0.98	0.87
OLSLR vs M-robust	0.88	0.97	0.96	0.97	0.80	0.90	0.98	0.87
OLSLR vs Quad	<0.001*	<0.001*	0.019*	<0.001*	0.056	0.042*	0.98	0.56
OLSLR vs Logist	0.88	0.97	0.96	0.97	0.86	0.90	0.98	0.87
M-robust vs Exp	0.88	0.97	0.96	0.97	0.63	0.90	0.98	0.87
M-robust vs Quad	<0.001*	<0.001*	0.019*	<0.001*	0.10	0.061	0.98	0.56
M-robust vs Logist	0.88	0.97	0.96	0.97	0.67	0.90	0.98	0.95
Exp vs Quad	<0.001*	<0.001*	0.019*	<0.001*	0.056	0.042*	0.98	0.65
Exp vs Logist	0.88	0.97	0.96	0.97	0.86	0.90	0.98	0.87
Quad vs Logist	<0.001*	<0.001*	0.019*	<0.001*	0.056	0.042*	0.98	0.56

AE: absolute error, VF: visual field, OLSLR: ordinary least squares linear regression, Exp: exponential regression, M-robust: M-estimator robust linear regression, Quad: quadratic regression, Logist: logistic regression, * represents $p < 0.05$

Supplemental Table 5. The AEs of second future MS prediction with each model.

Predicted VF	7	8	9	10	11	12	13
The number of VFs for the prediction	1-5	1-6	1-7	1-8	1-9	1-10	1-11
OLSLR	1.21 ± 0.99	1.25 ± 1.21	0.99 ± 0.91	1.05 ± 1.04	0.98 ± 0.94	1.10 ± 1.15	0.91 ± 0.87
Exp	1.18 ± 1.00	1.22 ± 1.22	0.96 ± 0.87	1.00 ± 1.03	0.93 ± 0.94	1.07 ± 1.15	0.91 ± 0.91
M-robust	1.20 ± 1.00	1.27 ± 1.22	0.99 ± 0.92	1.07 ± 1.05	1.00 ± 0.94	1.11 ± 1.13	0.91 ± 0.9
Quad	2.32 ± 2.04	2.11 ± 2.09	1.71 ± 1.68	1.66 ± 1.5	1.48 ± 1.33	1.37 ± 1.52	1.20 ± 1.04
Logist	1.19 ± 0.99	1.23 ± 1.22	0.97 ± 0.92	1.02 ± 1.05	0.95 ± 0.94	1.07 ± 1.14	0.90 ± 0.89
OLSLR vs Exp	0.89	0.85	0.89	0.63	0.66	0.91	0.98
OLSLR vs M-robust	0.89	0.85	0.89	0.76	0.75	0.99	0.98
OLSLR vs Quad	<0.001*	<0.001*	<0.001*	<0.001*	<0.001*	0.066	0.0061*
OLSLR vs Logist	0.89	0.85	0.89	0.76	0.75	0.91	0.98
M-robust vs Exp	0.89	0.85	0.89	0.57	0.52	0.91	0.98
M-robust vs Quad	<0.001*	<0.001*	<0.001*	<0.001*	<0.001*	0.070	0.0061*
M-robust vs Logist	0.98	0.85	0.89	0.63	0.6	0.91	0.98
Exp vs Quad	<0.001*	<0.001*	<0.001*	<0.001*	<0.001*	0.062	0.0061*
Exp vs Logist	0.89	0.89	0.89	0.76	0.82	0.99	0.98
Quad vs Logist	<0.001*	<0.001*	<0.001*	<0.001*	<0.001*	0.062	0.0061*

AE: absolute error, VF: visual field, OLSLR: ordinary least squares linear regression, Exp: exponential regression, M-robust: M-estimator robust linear regression, Quad: quadratic regression, Logist: logistic regression, * represents $p < 0.05$

Supplemental Table 6. The AEs of third future MS prediction with each model.

Predicted VF	8	9	10	11	12	13
The number of VFs for the prediction	1-5	1-6	1-7	1-8	1-9	1-10
OLSLR	1.44 ± 1.37	1.36 ± 1.26	1.32 ± 1.27	1.09 ± 1.04	1.27 ± 1.31	1.07 ± 1.09
Exp	1.39 ± 1.43	1.31 ± 1.25	1.26 ± 1.22	1.03 ± 1.04	1.22 ± 1.29	1.04 ± 1.10
M-robust	1.45 ± 1.37	1.38 ± 1.29	1.31 ± 1.28	1.11 ± 1.09	1.28 ± 1.33	1.07 ± 1.07
Quad	3.41 ± 2.99	2.97 ± 2.77	2.57 ± 2.61	2.24 ± 2.05	1.98 ± 1.90	1.71 ± 1.63
Logist	1.41 ± 1.41	1.33 ± 1.29	1.28 ± 1.26	1.05 ± 1.04	1.24 ± 1.29	1.04 ± 1.09
OLSLR vs Exp	0.85	0.80	0.72	0.63	0.90	0.82
OLSLR vs M-robust	0.94	0.81	0.90	0.83	0.95	0.99
OLSLR vs Quad	<0.001*	<0.001*	<0.001*	<0.001*	<0.001*	<0.001*
OLSLR vs Logist	0.85	0.81	0.78	0.70	0.90	0.82
M-robust vs Exp	0.85	0.80	0.75	0.63	0.90	0.82
M-robust vs Quad	<0.001*	<0.001*	<0.001*	<0.001*	<0.001*	<0.001*
M-robust vs Logist	0.85	0.80	0.78	0.63	0.90	0.82
Exp vs Quad	<0.001*	<0.001*	<0.001*	<0.001*	<0.001*	<0.001*
Exp vs Logist	0.85	0.81	0.78	0.83	0.95	0.99
Quad vs Logist	<0.001*	<0.001*	<0.001*	<0.001*	<0.001*	<0.001*

AE: absolute error, VF: visual field, OLSLR: ordinary least squares linear regression, Exp: exponential regression, M-robust: M-estimator robust linear regression, Quad: quadratic regression, Logist: logistic regression, * represents $p < 0.05$

Supplemental Table 7. The MAEs of first future VF prediction in each subgroup.

	Predicted VF	6	7	8	9	10	11	12	13
Subgroup	The number of VFs for the prediction	1-5	1-6	1-7	1-8	1-9	1-10	1-11	1-12
Early-to-moderate	OLSLR	2.95 ± 0.92	2.81 ± 0.92	2.71 ± 0.84	2.74 ± 0.89	2.73 ± 0.8	2.68 ± 1.3	2.72 ± 1.19	2.56 ± 0.91
	M-robust	2.95 ± 0.94	2.76 ± 0.94	2.63 ± 0.83	2.68 ± 0.91	2.65 ± 0.81	2.62 ± 1.28	2.69 ± 1.24	2.53 ± 0.96
	OLSLR vs M-robust	1.00	0.31	0.21	0.21	0.10	0.25	0.58	0.61
Advanced	OLSLR	2.42 ± 1.13	2.29 ± 1.16	2.19 ± 1.06	2.02 ± 0.93	2.01 ± 1.43	1.94 ± 0.98	1.87 ± 0.91	1.84 ± 0.93
	M-robust	2.35 ± 1.11	2.21 ± 1.15	2.09 ± 1.02	1.91 ± 0.95	1.92 ± 1.44	1.84 ± 0.87	1.76 ± 0.83	1.75 ± 0.91
	OLSLR vs M-robust	0.31	0.12	0.18	0.23	0.19	0.13	0.11	0.17
Stable	OLSLR	2.38 ± 1.17	2.24 ± 1.07	2.24 ± 1.12	2.04 ± 0.89	2.03 ± 0.94	1.97 ± 0.81	1.95 ± 0.87	1.93 ± 0.92
	M-robust	2.34 ± 1.15	2.16 ± 1.05	2.15 ± 1.08	1.94 ± 0.9	1.93 ± 0.92	1.89 ± 0.8	1.86 ± 0.86	1.86 ± 0.93
	OLSLR vs M-robust	0.60	0.16	0.20	0.10	0.13	0.16	0.17	0.16
Progressive	OLSLR	2.89 ± 0.92	2.77 ± 1.06	2.58 ± 0.84	2.6 ± 0.99	2.59 ± 1.45	2.53 ± 1.4	2.5 ± 1.26	2.35 ± 1.00
	M-robust	2.85 ± 0.95	2.72 ± 1.07	2.5 ± 0.85	2.53 ± 1.02	2.53 ± 1.47	2.44 ± 1.32	2.44 ± 1.27	2.29 ± 1.04
	OLSLR vs M-robust	0.51	0.20	0.066	0.38	0.19	0.21	0.62	0.37

MAE: mean absolute error, VF: visual field, OLSLR: ordinary least squares linear regression, M-robust: M-estimator robust linear regression

Supplemental Table 8. The MAEs of second future VF prediction in each subgroup.

	Predicted VF	7	8	9	10	11	12	13
Subgroup	The number of VFs for the prediction	1-5	1-6	1-7	1-8	1-9	1-10	1-11
Early-to-moderate	OLSLR	3.41 ± 1.15	3.21 ± 1.06	3.07 ± 0.98	2.98 ± 0.85	3.08 ± 1.47	3.01 ± 1.44	2.78 ± 1.02
	M-robust	3.39 ± 1.16	3.14 ± 1.08	3.01 ± 0.97	2.91 ± 0.84	3.02 ± 1.45	2.95 ± 1.44	2.75 ± 1.07
	OLSLR vs M-robust	0.70	0.22	0.35	0.13	0.32	0.39	0.62
Advanced	OLSLR	2.8 ± 1.38	2.65 ± 1.41	2.33 ± 1.11	2.23 ± 1.49	2.10 ± 0.93	2.09 ± 1.11	2.00 ± 1.04
	M-robust	2.71 ± 1.32	2.55 ± 1.39	2.22 ± 1.10	2.13 ± 1.51	2.00 ± 0.92	1.97 ± 0.96	1.89 ± 0.99
	OLSLR vs M-robust	0.20	0.21	0.23	0.21	0.18	0.14	0.18
Stable	OLSLR	2.76 ± 1.30	2.69 ± 1.43	2.33 ± 1.06	2.24 ± 1.02	2.23 ± 0.95	2.14 ± 0.97	2.06 ± 0.98
	M-robust	2.70 ± 1.28	2.59 ± 1.39	2.23 ± 1.04	2.11 ± 0.99	2.12 ± 0.93	2.04 ± 0.96	1.98 ± 1.00
	OLSLR vs M-robust	0.45	0.22	0.18	0.07	0.17	0.18	0.15
Progressive	OLSLR	3.36 ± 1.27	3.08 ± 1.14	2.95 ± 1.10	2.85 ± 1.49	2.80 ± 1.49	2.81 ± 1.55	2.60 ± 1.16
	M-robust	3.29 ± 1.25	3 ± 1.16	2.86 ± 1.09	2.80 ± 1.51	2.73 ± 1.48	2.72 ± 1.46	2.53 ± 1.14
	OLSLR vs M-robust	0.31	0.17	0.37	0.49	0.45	0.58	0.40

MAE: mean absolute error, VF: visual field, OLSLR: ordinary least squares linear regression, M-robust: M-estimator robust linear regression

Supplemental Table 9. The MAEs of third future VF prediction in each subgroup.

	Predicted VF	8	9	10	11	12	13
Subgroup	The number of VFs for the prediction	1-5	1-6	1-7	1-8	1-9	1-10
Early-to-moderate	OLSLR	3.89 ± 1.23	3.68 ± 1.17	3.32 ± 1.00	3.34 ± 1.53	3.46 ± 1.62	3.11 ± 1.28
	M-robust	3.85 ± 1.23	3.62 ± 1.20	3.24 ± 0.99	3.27 ± 1.48	3.36 ± 1.62	3.05 ± 1.29
	OLSLR vs M-robust	0.51	0.22	0.14	0.27	0.18	0.44
Advanced	OLSLR	3.13 ± 1.59	2.82 ± 1.40	2.64 ± 1.72	2.38 ± 1.02	2.28 ± 1.07	2.27 ± 1.32
	M-robust	3.02 ± 1.55	2.69 ± 1.39	2.52 ± 1.74	2.26 ± 1.01	2.17 ± 1.06	2.15 ± 1.16
	OLSLR vs M-robust	0.18	0.25	0.19	0.17	0.24	0.18
Stable	OLSLR	3.26 ± 1.59	2.85 ± 1.33	2.64 ± 1.26	2.50 ± 1.04	2.41 ± 1.14	2.30 ± 1.11
	M-robust	3.17 ± 1.54	2.73 ± 1.31	2.51 ± 1.23	2.36 ± 0.98	2.28 ± 1.10	2.20 ± 1.11
	OLSLR vs M-robust	0.40	0.16	0.080	0.08	0.15	0.089
Progressive	OLSLR	3.64 ± 1.37	3.50 ± 1.34	3.21 ± 1.65	3.07 ± 1.54	3.13 ± 1.63	2.95 ± 1.51
	M-robust	3.56 ± 1.39	3.42 ± 1.38	3.13 ± 1.69	3.01 ± 1.53	3.06 ± 1.63	2.85 ± 1.38
	OLSLR vs M-robust	0.22	0.40	0.37	0.53	0.69	0.34

MAE: mean absolute error, VF: visual field, OLSLR: ordinary least squares linear regression, M-robust: M-estimator robust linear regression
